# 1,1′-(Ethane-1,2-di­yl)dipyridinium bis­(iodate)

**DOI:** 10.1107/S1600536811020927

**Published:** 2011-06-11

**Authors:** Mostafa Gholizadeh, Behrooz Maleki, Mehrdad Pourayoubi, Mehdi Kia, Behrouz Notash

**Affiliations:** a Department of Chemistry, Ferdowsi University of Mashhad, Mashhad 91779, Iran; b Department of Chemistry, Sabzevar Tarbiat Moallem University, Sabzevar, Iran; c Chemistry Department, Shahid Beheshti University, G. C. Evin, Tehran 1983963113, Iran

## Abstract

The title salt, C_12_H_14_N_2_^2+^·2IO_3_^−^, exhibits two crystallographically independent iodate anions, the I atoms of which are each in a trigonal–pyramidal environment. In the dication, the two pyridine rings adopt an *anti* conformation with respect to each other; the angle between these two rings is 3.84 (19)°. In the crystal structure, C—H⋯O hydrogen bonds between the cations and anions lead to the formation of layers arranged parallel to the *ab* plane. I⋯O halogen bonds [*R*_2_^2^(4) graph-set motif] range between 2.873 (2) and 3.036 (3) Å and connect neighbouring IO_3_^−^ anions with each other along [100], so as to create a three-dimensional network.

## Related literature

For background about the oxidative properties of the iodate anion, see: Tamami *et al.* (2003 ▶); Singh *et al.* (2008 ▶). For related structures, see: Gholizadeh *et al.* (2011 ▶); Petrosyan *et al.* (1999 ▶, 2000 ▶). For graph-set analysis of hydrogen bonds, see: Bernstein *et al.* (1995 ▶). For the Cambridge Structural Database, see: Allen (2002 ▶).
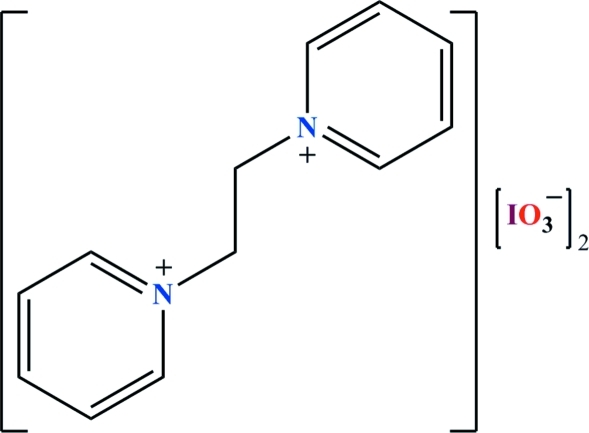


## Experimental

### 

#### Crystal data

C_12_H_14_N_2_^2+^·2IO_3_^−^
*M* *_r_* = 536.05
Monoclinic, 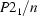

*a* = 7.9357 (4) Å
*b* = 10.2310 (4) Å
*c* = 18.6041 (9) Å
β = 91.017 (4)°
*V* = 1510.23 (12) Å^3^
*Z* = 4
Mo *K*α radiation
μ = 4.20 mm^−1^
*T* = 298 K
0.34 × 0.24 × 0.23 mm

#### Data collection

Stoe IPDS II diffractometer
Absorption correction: numerical [shape of crystal determined optically (*X-RED* and *X-SHAPE*; Stoe & Cie, 2005 ▶)]*T*_min_ = 0.310, *T*_max_ = 0.379
10467 measured reflections
4032 independent reflections
3081 reflections with *I* > 2σ(*I*)
*R*_int_ = 0.036

#### Refinement

*R*[*F* ^2^ > 2σ(*F* ^2^)] = 0.027
*wR*(*F* ^2^) = 0.054
*S* = 1.01
4032 reflections
199 parameters
H-atom parameters constrained
Δρ_max_ = 0.72 e Å^−3^
Δρ_min_ = −0.74 e Å^−3^

### 

Data collection: *X-AREA* (Stoe & Cie, 2005 ▶); cell refinement: *X-AREA*; data reduction: *X-AREA*; program(s) used to solve structure: *SHELXS97* (Sheldrick, 2008 ▶); program(s) used to refine structure: *SHELXL97* (Sheldrick, 2008 ▶); molecular graphics: *ORTEP-3 for Windows* (Farrugia, 1997 ▶) and *Mercury* (Macrae *et al.*, 2008) ▶; software used to prepare material for publication: *WinGX* (Farrugia, 1999 ▶).

## Supplementary Material

Crystal structure: contains datablock(s) I, global. DOI: 10.1107/S1600536811020927/zl2372sup1.cif

Structure factors: contains datablock(s) I. DOI: 10.1107/S1600536811020927/zl2372Isup2.hkl

Supplementary material file. DOI: 10.1107/S1600536811020927/zl2372Isup3.cml

Additional supplementary materials:  crystallographic information; 3D view; checkCIF report

## Figures and Tables

**Table 1:** Hydrogen-bond geometry (Å, °)

*D*—H⋯*A*	*D*—H	H⋯*A*	*D*⋯*A*	*D*—H⋯*A*
C1—H1⋯O3^i^	0.93	2.22	3.095 (5)	157
C3—H3⋯O2^ii^	0.93	2.37	3.133 (5)	139
C5—H5⋯O4^iii^	0.93	2.42	3.210 (5)	143
C6—H6*B*⋯O4^i^	0.97	2.37	3.230 (5)	148
C7—H7*A*⋯O2	0.97	2.52	3.420 (4)	154
C7—H7*B*⋯O1^i^	0.97	2.40	3.311 (5)	156
C8—H8⋯O1^i^	0.93	2.41	3.225 (5)	146
C9—H9⋯O1^iv^	0.93	2.49	3.297 (4)	146
C10—H10⋯O6^v^	0.93	2.18	3.051 (5)	156
C12—H12⋯O5^iii^	0.93	2.07	2.982 (5)	167

Symmetry codes: (i) 
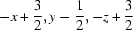
; (ii) 
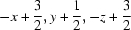
; (iii) 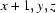; (iv) 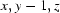; (v) 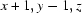.

**Fig. 1.**
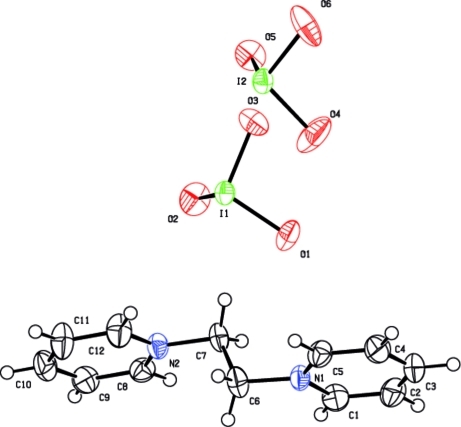
Molecular structure and atom labelling scheme for title salt with displacement ellipsoids drawn at the 50% probability level.

**Fig. 2.**
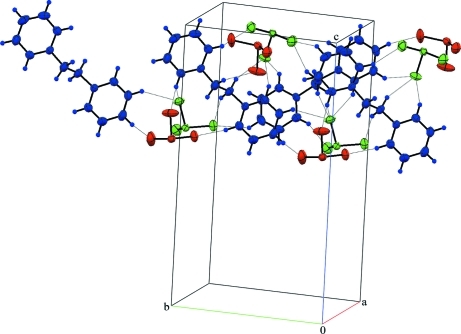
Part of the crystal structure of title compound with C—H···O hydrogen bonds shown as dotted lines. The two crystallographically independent iodate anions are shown in green and red colours, respectively, and the dication is coloured blue.

**Fig. 3.**
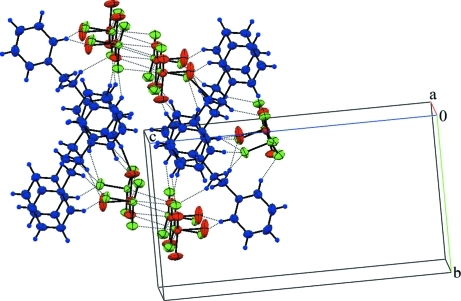
Part of the crystal packing showing the C—H···O hydrogen bonds and the I···O halogen bonds. The two crystallographically independent iodate anions are shown in green and red colours, respectively, and the dication is coloured blue.

## supplementary crystallographic information

### Comment

The iodate anion has the capability to oxidize various functional groups, so a range of salts of this anion, such as the potassium salt, have been prepared (Singh *et al.*, 2008). Iodate salts are also available supported on the commercial anionic resin Amberlyst A26, and also on poly(vinylpyridine) (Tamami *et al.*, 2003). In a recent publication, we described the synthesis and crystal structure of bis pyridinium 1,2-ethane periodate (Gholizadeh *et al.*, 2011). Here, we report the synthesis and single-crystal X-ray structure determination of the title iodate salt. Single crystals were obtained from CH_3_CN at room temperature. The asymmetric unit of the title salt, C_12_H_14_N_2_^2+^.2IO_3_^-^ (Fig. 1), contains two crystallographically independent iodate anions and one bis pyridinium 1,2-ethane dication. Each of the I atoms in the iodate anions adopts a trigonal pyramidal geometry, the bond angles at the I atoms vary in the range from 101.03 (12)° to 101.31 (13)° for I1 and 98.89 (14)° to 102.12 (18)° for I2. The I—O bond lengths (in the range from 1.779 (3) Å to 1.803 (3) Å) and the O—I—O bond angles are comparable to those in similar compounds like for example in [C_6_H_16_N_2_O_2_]^2+^[HI_2_O_6_]^1-^[IO_3_]^1-^ (Petrosyan *et al.*, 1999).

In the dication, the two pyridine moieties adopt an *anti* orientation with respect to one another. Some C—H···O hydrogen bonds, with C···O distances in the range from 2.888 (4) Å to 3.420 (4) Å, lead to a 2-D aggregation parallel to (001), Table 1 and Fig. 2. The I···O contacts (I1···O5^i^ = 2.873 (2) Å, O3—I1···O5^i^ = 163.07 (10)°, symmetry code: (i) 1 + *x*, *y*, *z*; O3···I2 = 2.961 (3) Å, I1—O3···I2 = 111.02 (13)°; I1···O5^ii^ = 3.000 (3) Å, O1—I1···O5^ii^ = 169.45 (11)°, symmetry code: (ii) 1 - *x*, 1 - *y*, 2 - *z*; I2···O2^ii^ = 3.036 (3) Å, I2···O2^ii^—I1^ii^ = 99.84°, symmetry code: (ii) 1 - *x*, 1 - *y*, 2 - *z*) involve IO_3_^-^ anions with each other, building *R*_2_^2^(4) rings along the *a* axis, which connect the two-dimensional structure into a three-dimensional network (Fig. 3). The I···O distances are comparable to those in similar iodate salts (CSD, Version 5.32, November 2010 update; Allen, 2002) *e.g.* in bis(β-alaninium) bis(iodate) monohydrate (CSD refcode ICAYAM; Petrosyan *et al.*, 2000). Consistent with the n-σ* character of the I···O halogen bond, the shortening of the I···O interaction distance leads to lengthening of the corresponding O—I covalent bond. The usual motif, which is found for iodate salts, is a four-membered ring containing two O and two I atoms and four I···O halogen bonds; however, a few other motifs are also found for crystal packing based on halogen bonds (CSD refcode ICAYEQ; Petrosyan *et al.*, 2000).

### Experimental

Preparation of bis pyridinium 1,2-ethane dibromide: 1,2-Dibromoethane (22 mmol) was added to pyridine (44.8 mmol) and dimethylformamide (40 ml) and refluxed. After 2 h, the precipitate was filtered and washed with diethyl ether and dried.

Preparation of title salt: To a solution of bis pyridinium 1,2-ethane dibromide (10 mmol) in H_2_O (25 ml), a solution of NaIO_3_ (20 mmol) in H_2_O (25 ml) was added and stirred. After 1 h, the precipitate was filtered and washed with H_2_O and crystallized from CH_3_CN at room temperature (yield: approximately 60%). ^1^H NMR (500.13 MHz, DMSO-d_6_, 300 K, TMS): 5.23 (*s*, 4H, 2CH_2_), 8.21 (*t*, 4H, Ar—H), 8.69 (*t*, 2H, Ar—H), 8.96 p.p.m. (*d*, 4H, Ar—H). ^13^C NMR (125.76 MHz, 300 K, TMS): 60.63, 129.36, 146.18, 147.54 p.p.m.

### Refinement

Carbon-bound H atoms were placed in calculated positions, C—H = 0.93 Å (aromatic) and 0.97 Å (CH_2_), and were included in the refinement using a riding model approximation, with *U*_iso_ = 1.2*U*_eq_(C).

### Crystal data

**Table:**

C_12_H_14_N_2_^2+^·2IO_3_^−^	*F*(000) = 1016
*M**_r_* = 536.05	*D*_x_ = 2.358 Mg m^−^^3^
Monoclinic, *P*2_1_/*n*	Melting point: 435 K
Hall symbol: -P 2yn	Mo *K*α radiation, λ = 0.71073 Å
*a* = 7.9357 (4) Å	Cell parameters from 4032 reflections
*b* = 10.2310 (4) Å	θ = 2.2–29.2°
*c* = 18.6041 (9) Å	µ = 4.20 mm^−^^1^
β = 91.017 (4)°	*T* = 298 K
*V* = 1510.23 (12) Å^3^	Prism, yellow
*Z* = 4	0.34 × 0.24 × 0.23 mm

### Data collection

**Table:**

Stoe IPDS II diffractometer	4032 independent reflections
Radiation source: fine-focus sealed tube	3081 reflections with *I* > 2σ(*I*)
graphite	*R*_int_ = 0.036
Detector resolution: 0.15 pixels mm^-1^	θ_max_ = 29.2°, θ_min_ = 2.2°
rotation method scans	*h* = −8→10
Absorption correction: numerical [shape of crystal determined optically (*X-RED* and *X-SHAPE*; Stoe & Cie, 2005)]	*k* = −12→14
*T*_min_ = 0.310, *T*_max_ = 0.379	*l* = −25→25
10467 measured reflections	

### Refinement

**Table:**

Refinement on *F*^2^	Primary atom site location: structure-invariant direct methods
Least-squares matrix: full	Secondary atom site location: difference Fourier map
*R*[*F*^2^ > 2σ(*F*^2^)] = 0.027	Hydrogen site location: inferred from neighbouring sites
*wR*(*F*^2^) = 0.054	H-atom parameters constrained
*S* = 1.01	*w* = 1/[σ^2^(*F*_o_^2^) + (0.0252*P*)^2^] where *P* = (*F*_o_^2^ + 2*F*_c_^2^)/3
4032 reflections	(Δ/σ)_max_ = 0.002
199 parameters	Δρ_max_ = 0.72 e Å^−^^3^
0 restraints	Δρ_min_ = −0.74 e Å^−^^3^

### Special details

**Table:**

Geometry. All e.s.d.'s (except the e.s.d. in the dihedral angle between two l.s. planes) are estimated using the full covariance matrix. The cell e.s.d.'s are taken into account individually in the estimation of e.s.d.'s in distances, angles and torsion angles; correlations between e.s.d.'s in cell parameters are only used when they are defined by crystal symmetry. An approximate (isotropic) treatment of cell e.s.d.'s is used for estimating e.s.d.'s involving l.s. planes.
Refinement. Refinement of *F*^2^ against ALL reflections. The weighted *R*-factor *wR* and goodness of fit *S* are based on *F*^2^, conventional *R*-factors *R* are based on *F*, with *F* set to zero for negative *F*^2^. The threshold expression of *F*^2^ > σ(*F*^2^) is used only for calculating *R*-factors(gt) *etc*. and is not relevant to the choice of reflections for refinement. *R*-factors based on *F*^2^ are statistically about twice as large as those based on *F*, and *R*-factors based on ALL data will be even larger.

### Fractional atomic coordinates and isotropic or equivalent isotropic displacement parameters (Å^2^)

**Table:**

	*x*	*y*	*z*	*U*_iso_*/*U*_eq_	
I1	0.83152 (3)	0.624927 (17)	0.927824 (10)	0.02673 (6)	
O1	0.8505 (4)	0.6730 (3)	0.83552 (12)	0.0460 (6)	
N1	1.0346 (4)	0.4963 (3)	0.69249 (13)	0.0308 (6)	
O2	0.7306 (4)	0.4690 (2)	0.91801 (14)	0.0494 (7)	
C5	1.0641 (5)	0.6193 (3)	0.71494 (18)	0.0369 (8)	
H5	1.1127	0.6341	0.7601	0.044*	
O3	0.6578 (4)	0.7251 (3)	0.95556 (14)	0.0479 (6)	
C1	0.9644 (5)	0.4709 (4)	0.62715 (17)	0.0392 (8)	
H1	0.9464	0.3852	0.6123	0.047*	
C7	0.9171 (5)	0.3298 (3)	0.77240 (18)	0.0371 (8)	
H7A	0.8658	0.3941	0.8035	0.045*	
H7B	0.8373	0.3088	0.7340	0.045*	
C2	0.9203 (5)	0.5730 (4)	0.58323 (18)	0.0483 (10)	
H2	0.8709	0.5568	0.5384	0.058*	
C6	1.0776 (5)	0.3854 (3)	0.7409 (2)	0.0412 (8)	
H6A	1.1523	0.4151	0.7793	0.049*	
H6B	1.1354	0.3180	0.7142	0.049*	
C4	1.0226 (6)	0.7226 (4)	0.67134 (19)	0.0473 (10)	
H4	1.0442	0.8078	0.6863	0.057*	
C3	0.9487 (6)	0.6991 (4)	0.6051 (2)	0.0504 (10)	
H3	0.9182	0.7685	0.5754	0.061*	
N2	0.9590 (4)	0.2100 (3)	0.81418 (13)	0.0316 (6)	
C8	0.9223 (5)	0.0913 (3)	0.78694 (18)	0.0346 (7)	
H8	0.8662	0.0836	0.7428	0.042*	
C12	1.0371 (6)	0.2234 (4)	0.87829 (19)	0.0467 (10)	
H12	1.0576	0.3064	0.8969	0.056*	
C9	0.9685 (5)	−0.0187 (3)	0.82504 (18)	0.0404 (8)	
H9	0.9432	−0.1010	0.8066	0.048*	
C11	1.0868 (6)	0.1146 (4)	0.9166 (2)	0.0515 (11)	
H11	1.1442	0.1238	0.9603	0.062*	
C10	1.0514 (5)	−0.0078 (4)	0.8899 (2)	0.0442 (9)	
H10	1.0833	−0.0821	0.9155	0.053*	
I2	0.33160 (3)	0.593964 (19)	0.925200 (11)	0.02945 (6)	
O5	0.1485 (3)	0.4896 (2)	0.92384 (14)	0.0438 (6)	
O4	0.3671 (4)	0.6018 (4)	0.83116 (14)	0.0749 (12)	
O6	0.2414 (4)	0.7504 (3)	0.94274 (19)	0.0656 (9)	

### Atomic displacement parameters (Å^2^)

**Table:**

	*U*^11^	*U*^22^	*U*^33^	*U*^12^	*U*^13^	*U*^23^
I1	0.02842 (11)	0.02451 (10)	0.02716 (9)	−0.00238 (8)	−0.00252 (7)	0.00285 (7)
O1	0.0538 (17)	0.0565 (17)	0.0277 (11)	−0.0048 (13)	−0.0004 (11)	0.0123 (10)
N1	0.0380 (16)	0.0258 (13)	0.0286 (12)	0.0015 (11)	−0.0018 (11)	0.0048 (9)
O2	0.0594 (18)	0.0343 (13)	0.0544 (15)	−0.0173 (13)	−0.0005 (13)	−0.0029 (11)
C5	0.047 (2)	0.0340 (17)	0.0299 (16)	−0.0026 (15)	−0.0014 (14)	−0.0013 (12)
O3	0.0406 (16)	0.0450 (15)	0.0582 (16)	0.0126 (12)	−0.0007 (12)	−0.0046 (12)
C1	0.046 (2)	0.0359 (18)	0.0360 (17)	−0.0001 (16)	−0.0035 (15)	−0.0060 (13)
C7	0.044 (2)	0.0264 (16)	0.0404 (17)	0.0055 (15)	−0.0051 (15)	0.0099 (13)
C2	0.053 (3)	0.063 (3)	0.0291 (17)	0.007 (2)	−0.0044 (16)	0.0034 (16)
C6	0.041 (2)	0.0327 (18)	0.050 (2)	0.0020 (15)	−0.0076 (16)	0.0124 (14)
C4	0.068 (3)	0.0268 (17)	0.047 (2)	0.0056 (17)	0.0094 (19)	0.0022 (14)
C3	0.060 (3)	0.045 (2)	0.046 (2)	0.0159 (19)	0.0063 (18)	0.0194 (16)
N2	0.0399 (16)	0.0245 (13)	0.0303 (13)	0.0009 (11)	−0.0042 (11)	0.0013 (9)
C8	0.040 (2)	0.0324 (17)	0.0317 (16)	−0.0021 (14)	0.0018 (14)	−0.0041 (12)
C12	0.070 (3)	0.0299 (17)	0.0399 (18)	−0.0026 (18)	−0.0132 (18)	−0.0030 (14)
C9	0.051 (2)	0.0248 (16)	0.0453 (19)	−0.0029 (15)	0.0054 (16)	−0.0021 (13)
C11	0.069 (3)	0.041 (2)	0.043 (2)	−0.0026 (19)	−0.019 (2)	0.0085 (15)
C10	0.052 (2)	0.0284 (17)	0.053 (2)	0.0031 (16)	−0.0008 (17)	0.0099 (14)
I2	0.02761 (12)	0.03071 (11)	0.02985 (10)	−0.00204 (9)	−0.00418 (8)	0.00538 (7)
O5	0.0388 (15)	0.0344 (13)	0.0581 (15)	−0.0117 (11)	−0.0027 (11)	−0.0044 (11)
O4	0.054 (2)	0.142 (4)	0.0285 (14)	−0.014 (2)	−0.0028 (13)	0.0167 (15)
O6	0.063 (2)	0.0259 (14)	0.106 (2)	0.0098 (14)	−0.0290 (18)	−0.0010 (14)

### Geometric parameters (Å, °)

**Table:**

I1—O2	1.793 (2)	C4—C3	1.376 (5)
I1—O1	1.795 (2)	C4—H4	0.9300
I1—O3	1.801 (3)	C3—H3	0.9300
N1—C5	1.345 (4)	N2—C12	1.341 (4)
N1—C1	1.353 (4)	N2—C8	1.346 (4)
N1—C6	1.485 (4)	C8—C9	1.376 (5)
C5—C4	1.369 (5)	C8—H8	0.9300
C5—H5	0.9300	C12—C11	1.375 (5)
C1—C2	1.367 (5)	C12—H12	0.9300
C1—H1	0.9300	C9—C10	1.368 (5)
C7—N2	1.486 (4)	C9—H9	0.9300
C7—C6	1.522 (5)	C11—C10	1.375 (5)
C7—H7A	0.9700	C11—H11	0.9300
C7—H7B	0.9700	C10—H10	0.9300
C2—C3	1.371 (6)	I2—O4	1.779 (3)
C2—H2	0.9300	I2—O6	1.786 (3)
C6—H6A	0.9700	I2—O5	1.803 (3)
C6—H6B	0.9700		
			
O2—I1—O1	101.03 (12)	C5—C4—C3	119.3 (4)
O2—I1—O3	101.10 (14)	C5—C4—H4	120.3
O1—I1—O3	101.31 (13)	C3—C4—H4	120.3
C5—N1—C1	121.7 (3)	C2—C3—C4	119.7 (3)
C5—N1—C6	119.3 (3)	C2—C3—H3	120.1
C1—N1—C6	119.0 (3)	C4—C3—H3	120.1
N1—C5—C4	120.0 (3)	C12—N2—C8	121.4 (3)
N1—C5—H5	120.0	C12—N2—C7	118.5 (3)
C4—C5—H5	120.0	C8—N2—C7	120.2 (3)
N1—C1—C2	119.2 (3)	N2—C8—C9	119.3 (3)
N1—C1—H1	120.4	N2—C8—H8	120.3
C2—C1—H1	120.4	C9—C8—H8	120.3
N2—C7—C6	109.2 (3)	N2—C12—C11	120.1 (3)
N2—C7—H7A	109.8	N2—C12—H12	119.9
C6—C7—H7A	109.8	C11—C12—H12	119.9
N2—C7—H7B	109.8	C10—C9—C8	120.5 (3)
C6—C7—H7B	109.8	C10—C9—H9	119.8
H7A—C7—H7B	108.3	C8—C9—H9	119.8
C1—C2—C3	120.1 (3)	C10—C11—C12	119.7 (4)
C1—C2—H2	119.9	C10—C11—H11	120.2
C3—C2—H2	119.9	C12—C11—H11	120.2
N1—C6—C7	109.5 (3)	C9—C10—C11	119.0 (3)
N1—C6—H6A	109.8	C9—C10—H10	120.5
C7—C6—H6A	109.8	C11—C10—H10	120.5
N1—C6—H6B	109.8	O4—I2—O6	102.12 (18)
C7—C6—H6B	109.8	O4—I2—O5	98.89 (14)
H6A—C6—H6B	108.2	O6—I2—O5	101.98 (14)
			
C1—N1—C5—C4	0.1 (6)	C6—C7—N2—C12	74.0 (4)
C6—N1—C5—C4	−179.0 (4)	C6—C7—N2—C8	−104.5 (4)
C5—N1—C1—C2	−0.9 (6)	C12—N2—C8—C9	−1.2 (6)
C6—N1—C1—C2	178.2 (4)	C7—N2—C8—C9	177.2 (3)
N1—C1—C2—C3	0.7 (6)	C8—N2—C12—C11	2.3 (6)
C5—N1—C6—C7	104.7 (4)	C7—N2—C12—C11	−176.1 (4)
C1—N1—C6—C7	−74.4 (4)	N2—C8—C9—C10	−0.2 (6)
N2—C7—C6—N1	173.7 (3)	N2—C12—C11—C10	−2.1 (7)
N1—C5—C4—C3	0.9 (6)	C8—C9—C10—C11	0.4 (6)
C1—C2—C3—C4	0.3 (6)	C12—C11—C10—C9	0.7 (7)
C5—C4—C3—C2	−1.1 (6)		

### Hydrogen-bond geometry (Å, °)

**Table:**

*D*—H···*A*	*D*—H	H···*A*	*D*···*A*	*D*—H···*A*
C1—H1···O3^i^	0.93	2.22	3.095 (5)	157.
C3—H3···O2^ii^	0.93	2.37	3.133 (5)	139.
C5—H5···O1	0.93	2.56	2.888 (4)	101.
C5—H5···O4^iii^	0.93	2.42	3.210 (5)	143.
C6—H6B···O4^i^	0.97	2.37	3.230 (5)	148.
C7—H7A···O2	0.97	2.52	3.420 (4)	154.
C7—H7B···O1^i^	0.97	2.40	3.311 (5)	156.
C8—H8···O1^i^	0.93	2.41	3.225 (5)	146.
C9—H9···O1^iv^	0.93	2.49	3.297 (4)	146.
C10—H10···O6^v^	0.93	2.18	3.051 (5)	156.
C12—H12···O5^iii^	0.93	2.07	2.982 (5)	167.

Symmetry codes: (i) −*x*+3/2, *y*−1/2, −*z*+3/2; (ii) −*x*+3/2, *y*+1/2, −*z*+3/2; (iii) *x*+1, *y*, *z*; (iv) *x*, *y*−1, *z*; (v) *x*+1, *y*−1, *z*.
